# Leukotriene B_4_ Enhances NOD2-Dependent Innate Response against Influenza Virus Infection

**DOI:** 10.1371/journal.pone.0139856

**Published:** 2015-10-07

**Authors:** Manon Le Bel, Jean Gosselin

**Affiliations:** 1 Laboratory of Innate Immunology, CHU de Québec-Université Laval Research Center, Quebec, Canada; 2 Department of Molecular Medicine, Faculty of Medicine, Université Laval, Quebec, Canada; Louisiana State University, UNITED STATES

## Abstract

Leukotriene B_4_ (LTB_4_), a central mediator of inflammation, is well known for its chemoattractant properties on effectors cells of the immune system. LTB_4_ also has the ability to control microbial infection by improving host innate defenses through the release of antimicrobial peptides and modulation of intracellular Toll-like receptors (TLRs) expression in response to agonist challenge. In this report, we provide evidences that LTB_4_ acts on nucleotide-binging oligomerization domain 2 (NOD2) pathway to enhance immune response against influenza A infection. Infected mice receiving LTB_4_ show improved survival, lung architecture and reduced lung viral loads as compared to placebo-treated animals. NOD2 and its downstream adaptor protein IPS–1 have been found to be essential for LTB_4_-mediated effects against IAV infection, as absence of NOD2 or IPS–1 diminished its capacity to control viral infection. Treatment of IAV-infected mice with LTB_4_ induces an increased activation of IPS-1-IRF3 axis leading to an enhanced production of IFNβ in lungs of infected mice. LTB_4_ also has the ability to act on the RICK-NF-κB axis since administration of LTB_4_ to mice challenged with MDP markedly increases the secretion of IL–6 and TNFα in lungs of mice. TAK1 appears to be essential to the action of LTB_4_ on NOD2 pathway since pretreatment of MEFs with TAK1 inhibitor prior stimulation with IAV or MDP strongly abrogated the potentiating effects of LTB_4_ on both IFNβ and cytokine secretion. Together, our results demonstrate that LTB_4_, through its ability to activate TAK1, potentiates both IPS–1 and RICK axis of the NOD2 pathway to improve host innate responses.

## Introduction

LTB_4_ is an endogenous lipid mediator of inflammation well known for its implication in the inflammatory process through the recruitment of immune cells [1–3]. Previous studies however highlighted the prominent role of LTB_4_ in the modulation of innate immune response against microorganisms [4–6]. In this regard, LTB_4_ was shown as an effective mediator of host defense against a wide range of bacteria (reviewed in [7]). Additionally, *in vitro* as well as *in vivo* experiments have reported the ability of LTB_4_ to control infection caused by several viruses including Influenza A virus (IAV) (reviewed in [8]). In this regard, it was demonstrated that LTB_4_ treatment significantly prevents IAV replication *in vivo* through an up-regulated production of antimicrobial peptides including beta-defensin–3 and the mouse cathelicidin-related antimicrobial peptide by neutrophils [9]. In addition, LTB_4_ has the capacity to potentiate TLR-mediated response of neutrophils to various TLR agonists [10,11]. Such effects of LTB_4_ were relied on at least two mechanisms. Firstly, neutrophil treatment with LTB_4_ increases expression of intracellular TLR7, 8 and 9, thus enhancing the recognition of foreign RNA and DNA [10,11] and secondly, LTB_4_ can also act on key molecules of the TLR signaling cascade, particularly TAK1, to potentiate the secretion of cytokines following recognition of TLR ligands [11].

Influenza RNA is known to be sensed by three classes of innate immune receptors including TLR3 and TLR7 [12,13], the RIG-like helicase (RLH) [14,15] as well as the nucleotide-binding oligomerization domain (NOD)-like receptors (NLR), including NOD2 [16] and NLRP3 ([17,18], and reviewed in [19]). Activation of these receptors in response to the detection of viral RNA ultimately leads to the production of type-I interferon (IFN) and inflammatory cytokines through the activation of intracellular signaling molecules including IRFs and NF-κB. NOD2 was initially described as a cytosolic sensor of muramyl dipeptide (MDP). In fact, NOD2 responds to bacterial peptidoglycan through the adaptor protein kinase RICK (Rip-like interactive clarp kinase, also named Rip2, RipK2 or CARDIAK) that is essential for the activation of both NF-κB and the mitogen-activated protein kinase (MAPK) pathways [20,21], and for the transcription of cytokine genes. Recently, the classical function of NOD2 has been challenged by demonstrating that single-stranded (ss) RNA from various viruses, which do not contain peptidoglycan, also have the ability to activate NOD2. In these conditions, NOD2 signals through the adaptor protein IPS–1 (also known MAVS/VISA/Cardif) leading to IRF3-dependent production of type 1 IFN, a potent antiviral mediator [16]. According to the fact that we have highlighted the potentiating effect of LTB_4_ on TLR system, we thus hypothesized that LTB_4_ could also act on NOD2 signaling pathways and promote innate host defense against RNA virus such as IAV. In the present study, we have evaluated whether LTB_4_ may activate NOD2-associated innate immune response against IAV infection, and looked at the ability of LTB_4_ to also activate the NOD2-RICK axis following recognition of the synthetic ligand MDP. Our results demonstrate that NOD2 contributes to the antiviral action of LTB_4_ since its effects in controlling IAV infection were significantly impaired in NOD2- and IPS-1-deficient mice. Similarly, the potentiating effect of LTB_4_ on cytokine production in response to MDP was also observed, indicating that LTB_4_ can act on both IPS–1 and RICK axis to enhance the release of type 1 IFN and cytokines in response to NOD2 agonists. Thus, this study reinforces the concept that LTB_4_ can act as an ubiquitous activator of host immune response.

## Materials and Methods

### Mice

C57BL/6 wild type (WT) mice were purchased from Charles River (St-Constant, QC, Canada). NOD2 deficient (NOD2^-/-^) mice were purchased from Jackson Laboratory (Bar Harbor, Maine, USA) whereas IPS–1 deficient (IPS–1^-/-^) mice were kindly provided by Dr. S. Akira, Osaka University (Osaka, Japan). These mice were generated and maintained in a C57BL/6 background as described previously [22]. NOD2^-/-^ and IPS–1^-/-^ breeding colonies were established at the CHU de Quebec Research Center. Animals, from 4 to 6 week old, were acclimated to standard laboratory conditions. The Animal Care Ethics Committee of the Centre de recherche du CHU de Quebec–Université Laval approved all experimental procedures (approval number 12–141).

### Virus infection of mice and lung viral load assessment

Infections were performed using Influenza virus strain A/Puerto Rico/8/34 (H1N1). IAV was propagated and isolated from Madin-Darby canine kidney (MDCK) cells and titrated using standard plaque assay in MDCK cells as reported [9]. Animals were infected intranasally (i.n.), at day 0 of the protocols, with a sublethal dose of IAV (50 Plaque forming unit (PFU)) or otherwise indicated. We daily assessed the general health of the animal by monitoring their physical appearance, body weight and temperature. The endpoint used to determine when the animals should be euthanized refers to the degree of weight loss. In the case where mice had lost more than 20% of their initial weight, they were sacrificed by lethal dose of isoflurane inhalation. In survival experiments following IAV infection, animals are not subjected to pain or distress and we did not observe any unexpected death [23]. To determine lung viral loads, mice were sacrificed by lethal dose of isoflurane and lungs harvested at day 3, 5 and 7 post-infection. Viral burden was measured in lung homogenates using standard plaque assay in MDCK cells as described elsewhere [9].

### Treatment of mice with LTB_4_ and MDP

LTB_4_ was obtained as an ethanolic solution (Cayman Chemicals, Ann Arbor, MI, USA) and prepared by dilution of the ethanolic LTB_4_ in a saline solution containing 0.9% (w/v) NaCl [10]. Saline solution without LTB_4_ was used as a placebo. N-acetyl muramyl dipeptide (MDP) (InvivoGen, San Diego, CA, USA) was reconstituted with endotoxin-free water. Mice were daily treated intravenously (i.v.) with LTB_4_ (1 μg/kg), MDP (10 mg/kg) or with a placebo starting from day 1 to day 10 post-IAV infection.

### Measurement of IFNβ and cytokines in lungs of mice

IFNβ and cytokine levels were determined in lung homogenates of WT, NOD2^-/-^ and IPS–1^-/-^ mice infected or not with IAV (50 PFU) and treated with LTB_4_ (1μg/kg) or MDP (10 mg/kg) alone and in combination with LTB_4_. Lungs were harvested 6 hours following treatments and IFNβ (PBL interferon source, Piscataway, NJ, USA), TNFα and IL–6 (eBioscience, San Diego, CA, USA) levels were measured using specific ELISA kits.

### Histological analysis of lung sections

WT and NOD2^-/-^ mice were infected or not with IAV (50 PFU) and treated with LTB_4_. Animals were sacrificed at day 5 post-infection and lungs were harvested and fixed in paraformaldehyde (4%). Tissues were embedded in paraffin and coronal sections were stained with Hematoxylin and Eosin (H&E) for microscopic analyses [9].

### Western blot analysis

Western blot analyses were performed on protein samples extracted from lung homogenates of WT, NOD2^-/-^ and IPS–1^-/-^ mice, infected or not with IAV (50 PFU) and treated with LTB_4_ or MDP. Lungs were harvested 6 hours post-treatment and samples were homogenized in ice-cold cell lysis buffer (Cell Signaling Technology, Danvers, MA, USA) containing protease and phosphatase inhibitor cocktail (Roche Applied Science, Laval, QC, Canada). Protein concentrations were determined by the BCA assay (Pierce, Rockford, IL, USA). Equal amounts of protein (40 μg) were separated on SDS/10% PAGE, transferred onto PVDF membranes and immunoblotted overnight with selected anti-IPS–1 (Cell signaling, Danvers, MA, USA), anti-phospho-IRF7 (Ser471/472), anti-phospho-IRF3 (Ser396) (Bioss, Worburn, MA, USA) anti-IRF7, anti-IRF3 (Santa Cruz, Dallas, TX, USA), anti-NF-κB p65 (Millipore, Billerica, MA, USA) anti-phospho TAK1 (Thr–187) (cell Signaling Technology, Danvers, MA, USA) and anti-actin (BioLegend, San Diego, CA, USA) antibodies. Membranes were washed in Tris-buffered saline (TBS)-0.1% Tween 20 solution (Thermo Fisher Scientific, Rockford, IL, USA) prior to incubation with appropriate secondary horseradish peroxidase (HRP)-conjugated antibody (Jackson Immunoresearch, West Grove, PA, USA). HRP activity was revealed by incubation with the Clarity ECL substrate (Bio Rad, Mississauga, ON, Canada). Chemiluminescence reactions were visualized and quantitatively analyzed using Alphaview software (Alpha Innotech Corp., San Leandro, CA, USA).

### Murine embryonic fibroblast preparation and cell treatment

Murine embryonic fibroblasts (MEFs) were obtained from 13- to 14-days-old WT mouse embryos digested with 0.5% trypsin-EDTA solution for 30 minutes at 37°C [24]. MEFs were cultured in MEM supplemented with 20% heat inactivated FBS. Cells were pretreated for 30 minutes with specific TAK1 inhibitor 5Z-7-oxozeaenol (1 μM) (Sigma-Aldrich, Oakville, ON, Canada), infected with Influenza A virus at 0.5 multiplicity of infection (m.o.i.) and treated with placebo or LTB_4_ (1 μM). Cells were also stimulated with MDP alone (10 μg/ml) or in combination with LTB_4_ (1 μM). Supernatants and cells were collected 6 hours post-treatment. Protein samples were extracted from cells and assayed for western blot analyses, as detailed above, using anti-IPS–1, anti-phospho-IRF3, anti-phospho-IRF7 and anti-NF-κB p65 antibodies. Supernatants were assayed for IFNβ, TNFα and IL–6 by ELISA.

### Statistical analysis

Differences in group survival rates were compared using a log-rank test through the XLSTAT software (Addinsoft, New-York, NY, USA). All other experiments were analyzed by Student’s *t* test (two-tailed, unpaired) using the GraphPad Prism software program, version 5.00 (GraphPad Soft- ware, San Diego, CA, USA). Differences were considered significant at p≤0.05.

## Results

### NOD2 contributes to LTB_4_-mediated improvement of viral clearance in IAV-infected mice

**Fig 1:**
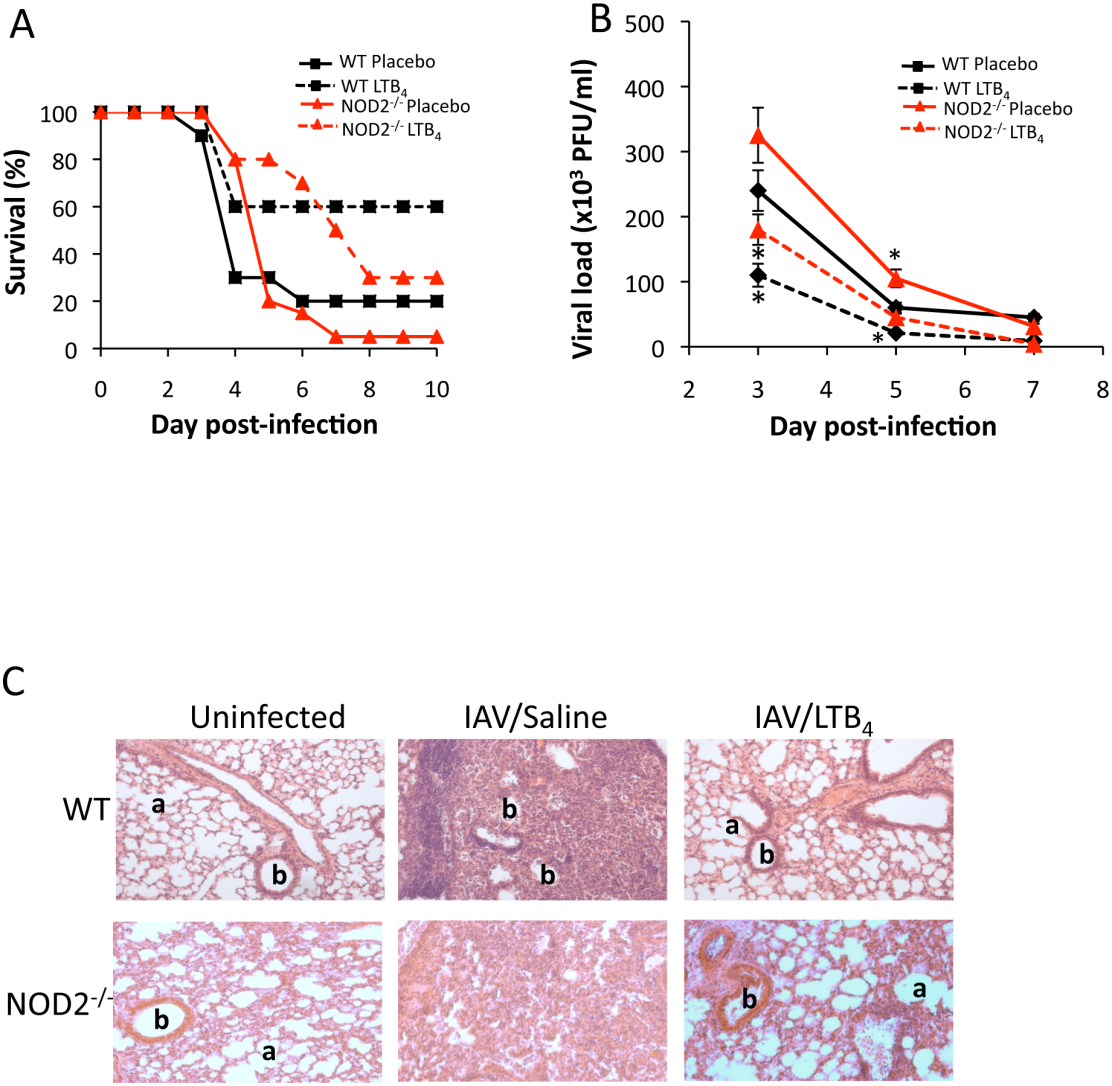
NOD2 contributes to the effects of LTB_4_ treatment on the control of IAV-infection. WT and NOD2^-/-^ mice (n = 6 per group) were infected with IAV and daily treated (i.v.) with placebo or LTB_4_ (1 μg/kg). **(A)** Mice were infected with a lethal dose of IAV (3000 PFU, i.n.) and survival was monitored daily for 10 days. **(B)** Mice were infected with IAV (50 PFU, i.n.) and lungs were harvested at days 3, 5 and 7 post-infection for viral load assessment by plaque assay. Data are representative of three independent experiments. *p<0.05 as compared to WT placebo-treated group. **(C)** Histological examination of mice infected with IAV (50 PFU, i.n.) and treated daily with LTB_4_ (i.v.). Five days post-infection, lungs were harvested and tissues were fixed and stained with hematoxylin-eosin for microscopic observation. a: alveolar and b: bronchiolar structure (original magnification ×100).

In the first set of experiments, we wanted to evaluate whether NOD2 pathway contributes to the LTB_4_-associated improvement of innate response against IAV infection. WT and NOD2^-/-^ mice were infected with a lethal dose of IAV (3000 PFU) and daily treated (i.v.) with LTB_4_ (1 μg/kg) or placebo from day 1 to 10 post-infection. As shown in Fig 1A, daily administration of LTB_4_ to WT mice infected with IAV results in a significant increase in survival compared to the placebo-treated animals. Such effect of LTB_4_ was reduced in NOD2^-/-^ mice, indicating that LTB_4_ may interact with NOD2 pathway to control viral infection. We next wanted to evaluate whether such protecting effect of LTB_4_ treatment on survival correlates with reduced viral loads in lungs of IAV-infected mice. For this experiment, mice were infected with sublethal dose of IAV (50 PFU) to ensure survival and to allow characterization of the effects of LTB_4_ on viral infection. LTB_4_ treatment significantly reduces viral loads in lungs of WT mice on day 3 and 5 post-infection as compared to the placebo groups (Fig 1B). We did not observe any significant difference in viral load at day 7 following infection since after this time, mice usually clear the virus completely [9]. Again such effect of LTB_4_ treatment on IAV viral loads was markedly affected by the lack of functional NOD2 receptor. The effects of LTB_4_ administration on survival of IAV-infected NOD2^-/-^ mice were however not entirely abolished compared to the placebo groups, suggesting that other innate sensors (such as TLRs) sensitive to LTB_4_ action may compensate for the absence of functional NOD2 [11]. Histological examination revealed a marked decreased in leukocyte infiltration and a reduced bronchiolar wall thickening and alveolar obstruction in the lung of LTB_4_-treated IAV-infected WT mice as compared to placebo-treated animals (Fig 1C). The effect of LTB_4_ on lung architecture improvement was however markedly reduced in NOD2^-/-^ mice infected with IAV, since the airway remodeling and prevention of cell infiltration are less effective in these animals, supporting the contribution of NOD2 in the protecting effect of LTB_4_ against IAV infection.

**Fig 2:**
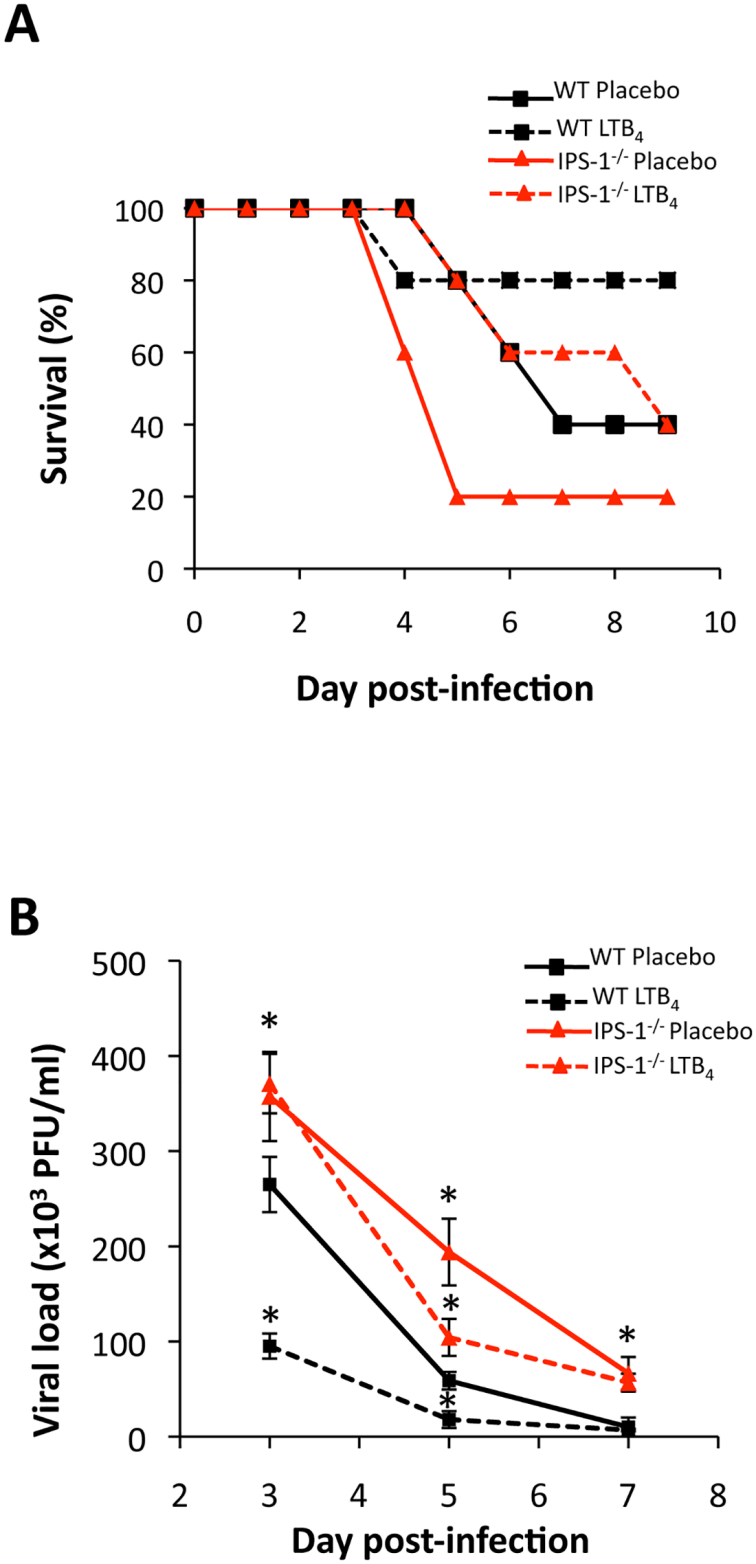
IPS–1 is required for LTB_4_ to control IAV infection. WT and IPS–1^-/-^ mice (n = 6 per group) were infected with IAV and daily treated (i.v.) with placebo or LTB_4_ (1 μg/kg). **(A)** For survival experiments, mice were infected with a lethal dose of IAV (3000 PFU, i.n.) and monitored daily for 10 days. **(B)** Mice were infected with IAV (50 PFU, i.n.) and lungs were harvested at days 3, 5 and 7 post-infection for viral load measurement. Data are representative of three independent experiments. *p<0.05 as compared to WT placebo-treated group.

Whereas RICK is a critical signaling intermediate in the NOD2 pathway following detection of bacterial component, viral infection rather triggered the NOD2-IPS–1 axis to elaborate effective immune response [14,16,25]. We therefore wanted to investigate whether IPS–1 is essential to LTB_4_-mediated potentiation of NOD2 antiviral response. As performed with NOD2^-/-^ mice, WT and IPS–1^-/-^ mice were infected with lethal or sublethal doses of IAV, daily treated with LTB_4_ or with a placebo, and survival rate as well as lung viral loads were monitored as above. First, we observed that IPS–1^-/-^ mice were more susceptible to IAV infection and that the effects of LTB_4_ were significantly affected in these animals (Fig 2A and 2B), indicating that LTB_4_ necessitates an interaction between IPS–1 and NOD2 to be fully effective.

### LTB_4_ promotes activation of IRF3 and NF-κB in IAV-infected mice

**Fig 3:**
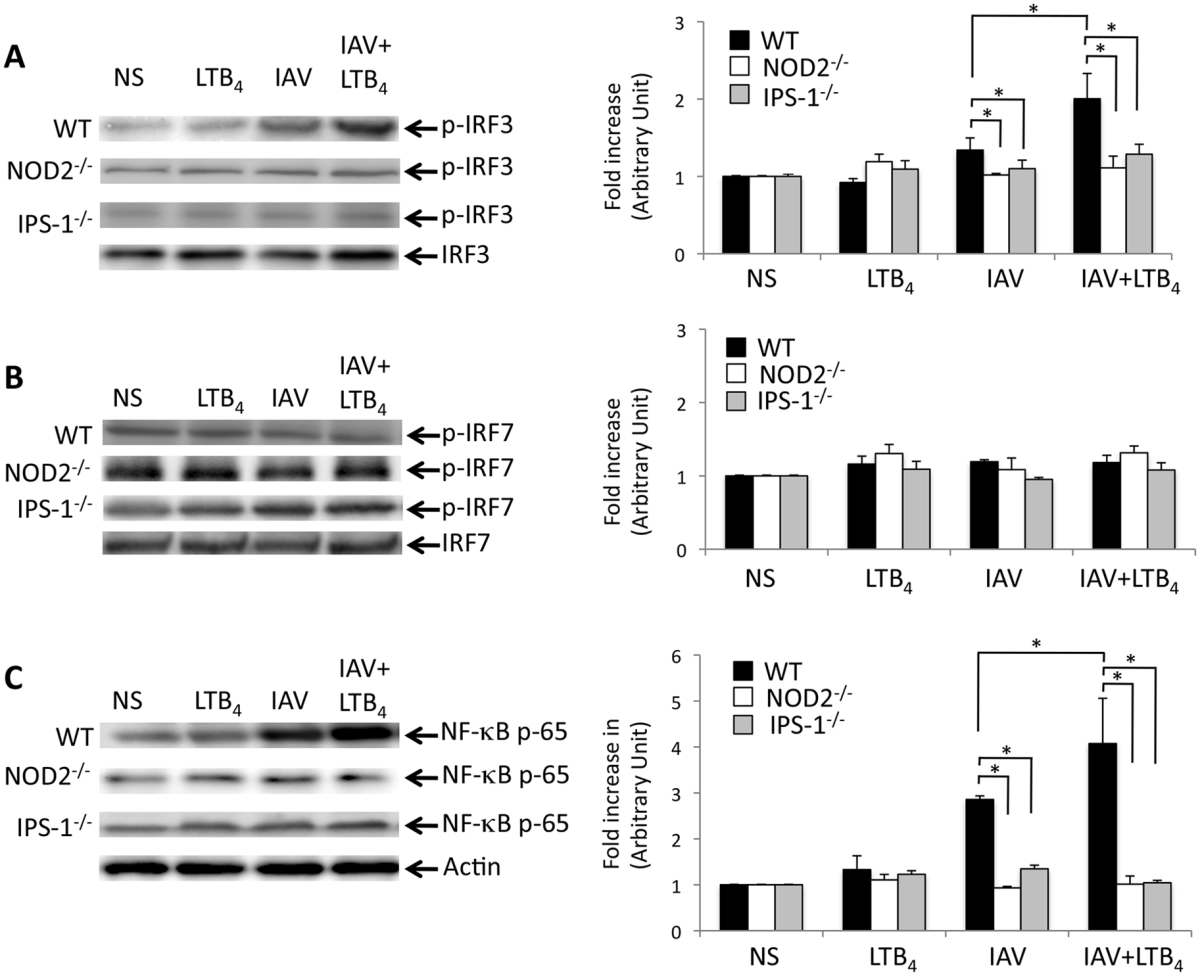
NOD2 is involved in the enhanced effect of LTB_4_ on activation of IRF3 and NF-κB in IAV-infected mice. WT, NOD2^-/-^ and IPS–1^-/-^ mice (n = 6 per group) were infected with IAV (50 PFU, i.n.) and treated (i.v.) with placebo or LTB_4_ (1 μg/kg). Six hours post-treatment, mice were sacrificed and lungs were homogenized for protein extraction. Immunoblots of **(A)** phosphorylated-IRF3 on serine 396 (p-IRF3), **(B)** phosphorylated IRF7 on serine 471/472 (p-IRF7) and **(C)** NF-κB-p65 proteins, as well as their respective loading control, in lung homogenates. Right panels show densitometric analysis of p-IRF3, p-IRF7 and NF-κB-p65 immunoblots. Fold increase in proteins expression is expressed relative to the respective not-stimulated (NS) group. Data are representative of three independent experiments. *p<0.05 as compared to the indicated groups.

Triggering of NOD2 by bacterial agonists leads to the translocation of NF-κB, whereas recognition of viral RNA induces the activation of IRF3 and IFN production [16,26]. While recognized to be involved in cytokine gene activation, NF-κB can contribute to induce type 1 IFN gene expression [27]. Thus, we have next evaluated whether treatment of IAV-infected mice with LTB_4_ may have consequences on IRF3, IRF7 and NF-κB activation following infection. We performed western blot analyses of phospho-IRF3 (p-IRF3), p-IRF7 and NF-κB (p–65) on protein samples from lung homogenates isolated from WT, NOD2^-/-^ and IPS–1^-/-^ mice infected with IAV and treated with LTB_4_. LTB_4_ alone did not influence the expression of IRF3 and IRF7 while it slightly enhances expression levels of p–65 (Fig 3A, 3B and 3C). As expected, IAV infection leads to a significant increase in the expression levels of p-IRF3 and NF-κB p65 but not p-IRF7. Interestingly, LTB_4_ administration to IAV-infected mice markedly enhanced phosphorylation of IRF3 and translocation of the p65 subunit of NF-κB. Such potentiating effect of LTB_4_ was strongly reduced in infected mice lacking NOD2 or IPS–1, thus supporting that LTB_4_ may act on signaling events of the NOD2-IPS–1 axis.

### NOD2-IPS–1 are required for LTB_4_-induced potentiation of IFNβ and cytokine secretion in IAV-infected mice

**Fig 4:**
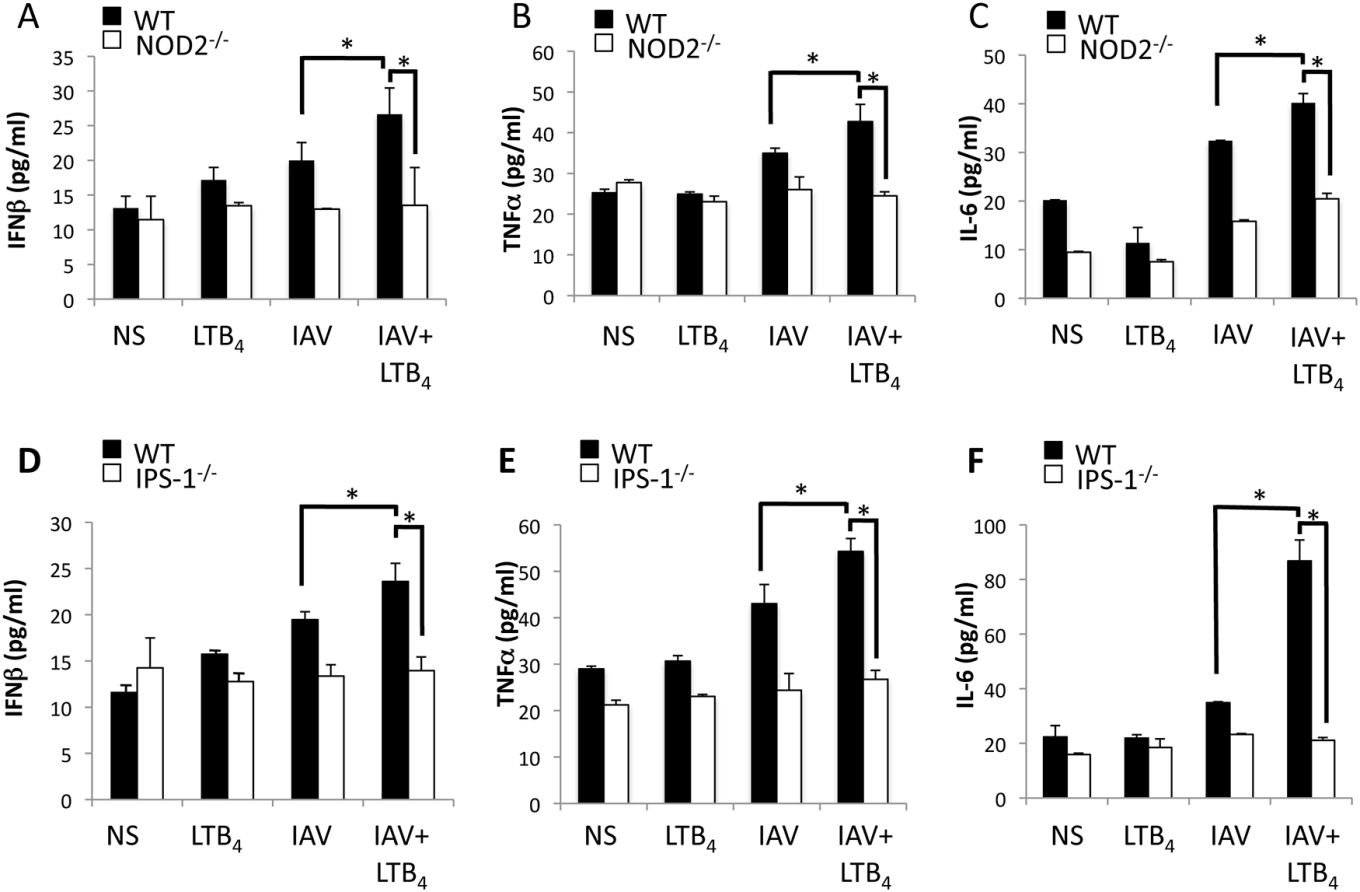
LTB_4_-mediated increased production of inflammatory mediators is abrogated in IAV-infected NOD2^-/-^ and IPS–1^-/-^ mice. WT, NOD2^-/-^ and IPS–1^-/-^ mice (n = 6 per group) were infected with IAV (50 PFU, i.n.) and treated (i.v.) daily with placebo or LTB_4_ (1 μg/kg). Mice were sacrificed 6 hours post-treatment and levels of **(A, D)** IFNβ, **(B, E)** TNFα and **(C, F)** IL–6 were determined in lung homogenates by ELISA. Data are representative of three independent experiments. *p<0.05 as compared to indicated groups.

To further validate the capacity of LTB_4_ to potentiate NOD2-IRF3 response to IAV infection, we have compared the concentrations of IFNβ released in lungs of IAV-infected mice treated with LTB_4_ or with a placebo. Considering that NOD2 triggering also results in the release of inflammatory cytokines [26], we have evaluated the production of TNFα and IL–6 following LTB_4_ treatment of IAV infected mice. While modest, IAV infection induces the production of IFNβ, TNFα and IL–6 in the lungs of IAV-infected WT mice as compared to uninfected animals (Fig 4). LTB_4_ alone did not induce secretion of IFNβ or inflammatory cytokines compared to unstimulated animals but when administered in mice infected with IAV, we measured a marked increase of IFNβ, TNFα and IL–6 concentrations in lungs of mice. The effects of LTB_4_ were significantly abrogated in the lungs of NOD2^-/-^ (Fig 4A, 4B and 4C) and IPS1^-/-^ (Fig 4D, 4E and 4F) animals, suggesting that its potentiating action on IFNβ and cytokine secretion necessitates activation of the NOD2-IPS–1 axis.

As previously stated, non-viral ligand sensed by NOD2 receptor triggers the RICK pathway that culminates in the production of inflammatory cytokines. As we have observed that LTB_4_ potentiates the NOD2-IPS–1 axis and that LTB_4_ treatment increases activation of NF-κB, we wanted next to determine whether LTB_4_ also have the ability to modulate the NOD2-RICK pathway. We treated WT and NOD2^-/-^ mice with the NOD2 ligand MDP alone or in combination with LTB_4_ and measured the lungs levels of TNFα and IL–6. As presented in Table 1, MDP alone did not significantly increase the secretion of TNFα or IL–6 in the lungs of WT mice compared to the control groups. However, a significant enhanced production of both cytokines was observed in the lung of WT mice treated with MDP in combination with LTB_4_. Such effects of LTB_4_ were totally abolished in NOD2^-/-^ mice. These results demonstrate that LTB_4_ can also interact with the NOD2-RICK pathways engaged following recognition of non-viral ligand like MDP.

**Table 1:** LTB_4_ potentiates *in vivo* secretion of TNFα and IL–6 in response to MDP treatment.

	**TNFα secretion**		
	**NS**	**MDP**	**MDP+LTB**_**4**_
**C57Bl/6**	17.9 ± 2.4	29.2 ± 4.1	57.5 * ± 3.7
**NOD2** ^**-/-**^	21.2 ± 3.8	19.3 ± 1.8	20.1 ± 2.5
	**IL–6 secretion**		
	**NS**	**MDP**	**MDP+LTB**_**4**_
**C57Bl/6**	20.2 ± 3.4	31.5 ± 6.5	61.6 * ± 7.6
**NOD2** ^**-/-**^	15.7 ± 4.7	19.9 ± 3.2	24.0 ± 4.9

WT and NOD2-/- (n = 6 per group) were treated with MDP (10 mg/kg) alone or in combination with LTB_4_ (1 μg/kg). Animals were sacrificed 6 hours post-treatment and levels of TNFα and IL-6 were measured in lung homogenates of mice by ELISA. Administration of LTB_4_ alone to naive mice did not induce significant production of TNFα and IL–6.

*p<0.05 as compared to respective MDP-treated group. NS: not stimulated.

### TAK1 is essential for NOD2-induced anti-IAV responses

**Fig 5:**
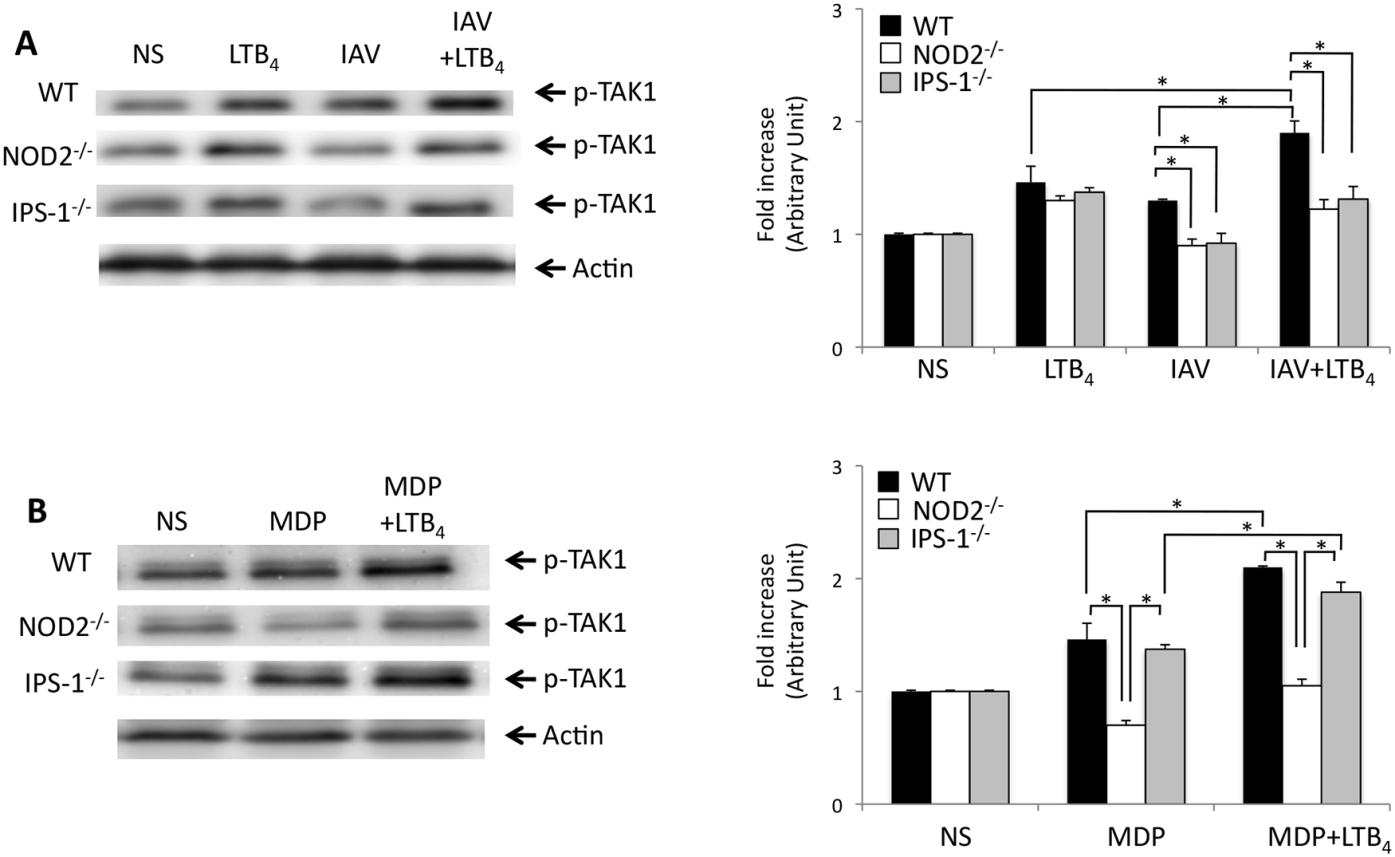
TAK1 activation is essential to the priming effect of LTB_4_ on both IPS–1 and RICK axis of the NOD2 pathway. WT, NOD2^-/-^ and IPS–1^-/-^ mice (n = 6 per group) were infected with IAV (50 PFU i.n.) or challenged with MDP (10 mg/kg) and treated (i.v.) with placebo or LTB_4_ (1 μg/kg). Six hours post-treatment, mice were sacrificed and lungs were homogenized for protein extraction. Representative immunoblots of phosphorylated TAK1 on threonine 187 and actin loading control in lung homogenates of **(A)** mice treated with LTB_4_ alone or infected with IAV and treated with LTB_4_ or a placebo, or **(B)** mice treated with MDP alone or in combination with LTB_4_. Right panels show densitometric analysis of p-TAK1 expression in lung homogenates. Fold increase in TAK1 is expressed relative to the respective not-stimulated (NS) group. Data are representative of two independent experiments. *p<0.05 as compared to the indicated groups.

As TAK1 has been proposed to contribute to NOD2-RICK signaling pathway when activated with non-viral ligands [28,29] and that TAK1 can be activated by LTB_4_ under distinct conditions [10,11], we next evaluated whether TAK1 is also required by NOD2-mediated activation of IPS-1-IRF3 axis to produce type 1 IFN. We have first compared the phosphorylation levels of TAK1 in lungs of WT, NOD2^-/-^ and IPS–1^-/-^ mice treated with LTB_4_ alone, to those of mice infected with IAV, treated or not with LTB_4_. LTB_4_ alone gives rise to an increased phosphorylation level of TAK1 in WT mice which was also observed, to a lesser extent, following IVA infection (Fig 5A). When LTB_4_ was administered to IAV-infected mice, increased levels of phosphorylated TAK1 were observed. Such effects of LTB_4_ were reduced in IAV-infected NOD2^-/-^ and IPS–1^-/-^ mice. The lack of functional NOD2 receptor or IPS–1 adaptor protein did not however alter the effect of LTB_4_ alone on TAK1 phosphorylation. Next, we looked for the effects of LTB_4_ on TAK phosphorylation in mice treated with MDP. Treatment with MDP alone induces an increased phosphorylation of TAK1 in WT mice, which fails in NOD2^-/-^ mice but not in IPS–1^-/-^ animals (Fig 5B). Concomitant administration of LTB_4_ to MDP challenged WT mice potentiates TAK1 phopsphorylation which was affected by the absence of NOD2. Together, these results indicate that TAK1 contributes to NOD2-mediated innate response following activation by both a viral and a non-viral ligands and that LTB_4_ may directly induce phosphorylation of TAK1 to enhance NOD2-mediated immune responses.

**Fig 6:**
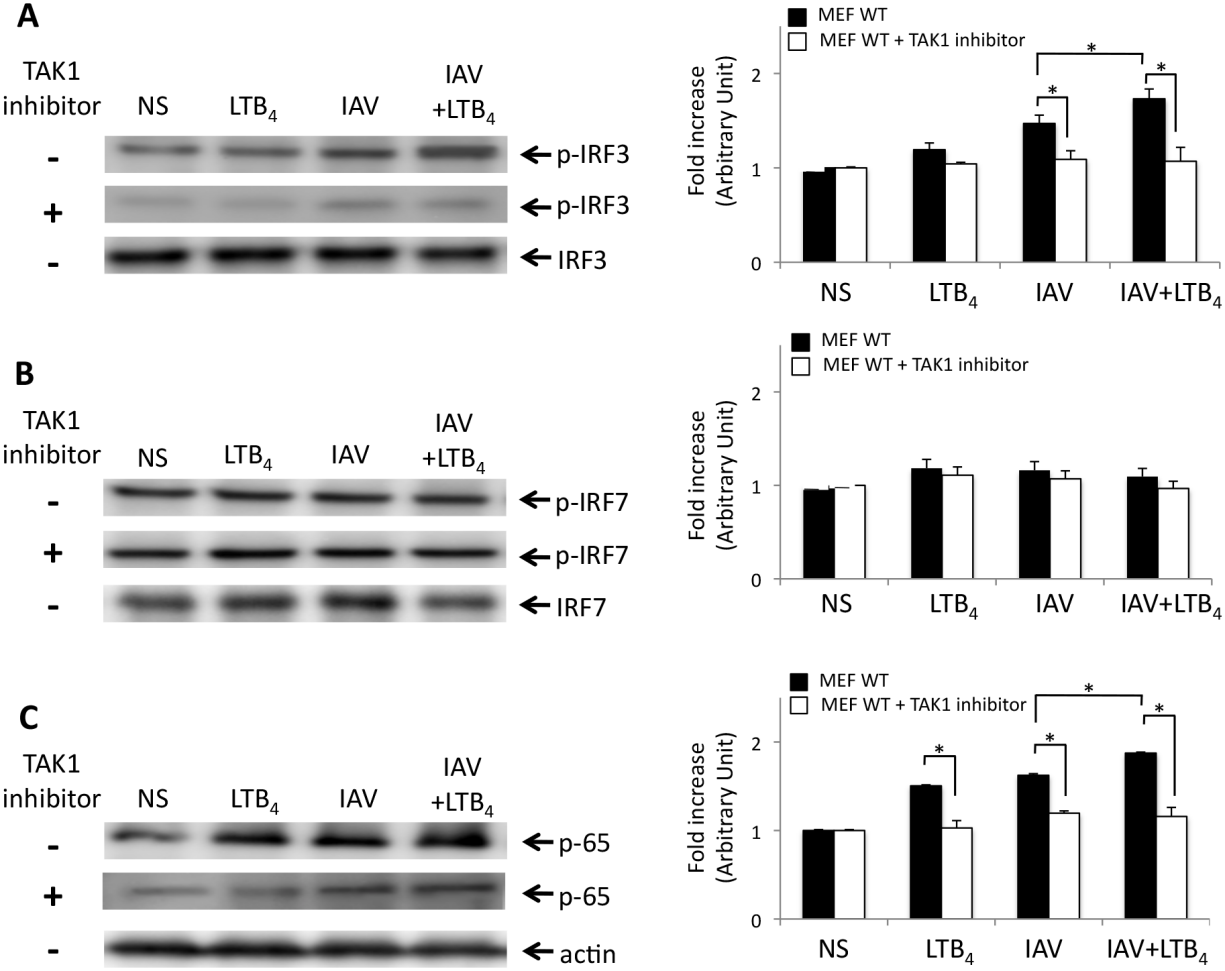
TAK1 is required for LTB_4_ to enhance activation of IRF3 and NF-κB. WT Mouse Embryonic Fibroblasts (MEFs) were treated with specific TAK1 inhibitor 5Z-7-oxozeaenol (1 μM) 30 minutes prior to IAV infection (0.5 m.o.i) and LTB_4_ treatment (1 μM). Cells were harvested 6 hours post-treatment and proteins were extracted for western blot analyses. Representative immunoblots of **(A)** phosphorylated-IRF3 on serine 396 (p-IRF3) **(B)** phosphorylated IRF7 on serine 471/472 (p-IRF7) and **(C)** NF-κB-p65 proteins and their respective IRF3, IRF7 and actin loading control in MEFs. Right panels show the folds increase in p-IRF3, p-IRF7 and NF-κB-p65 expression in MEFs. Fold increase in proteins expression is expressed relative to the respective not-stimulated (NS) group. Data are representative of two independent experiments. *p<0.05 as compared to the indicated groups.

**Fig 7:**
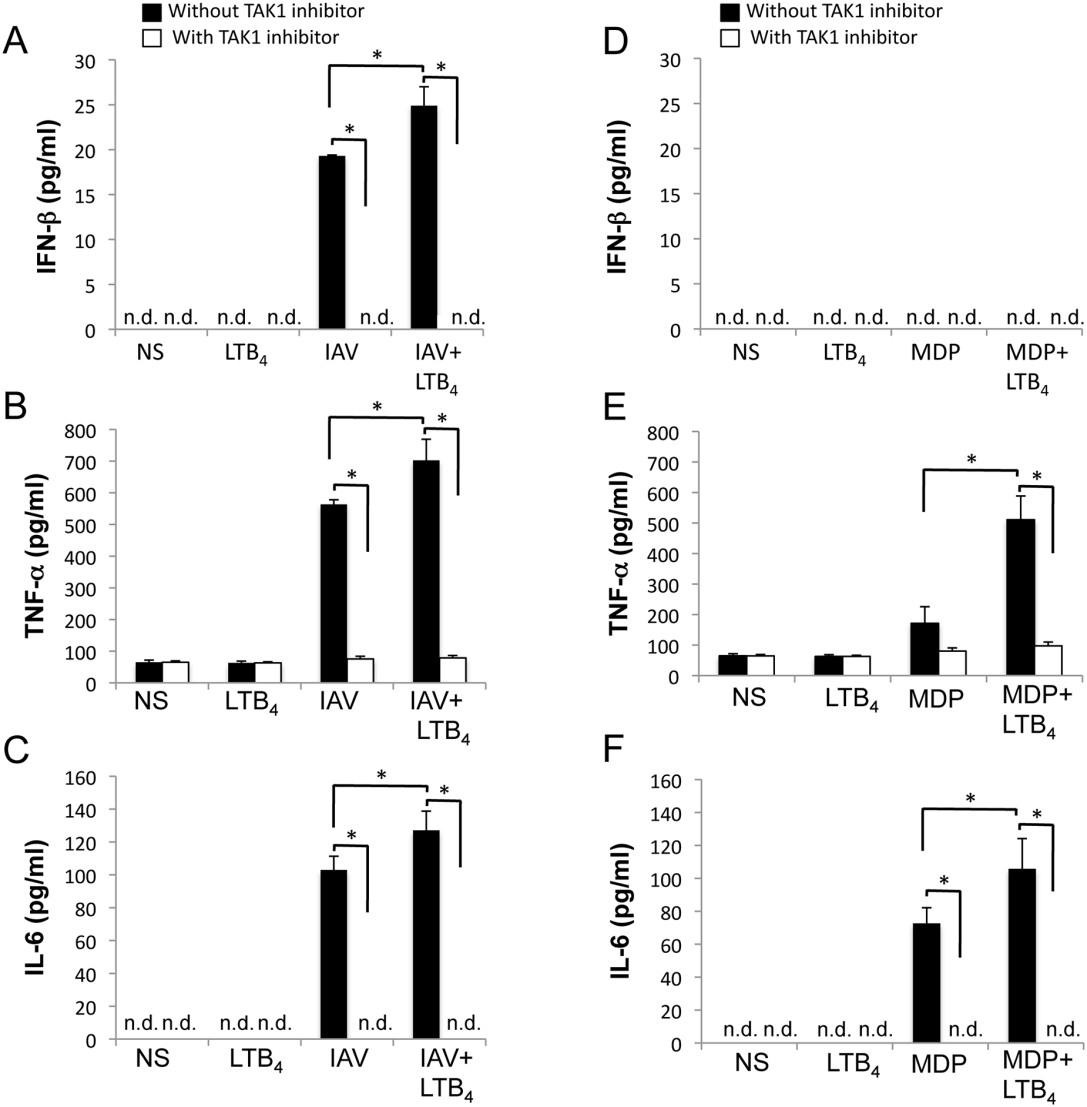
TAK1 contributes to the potentiating effect of LTB_4_ on the release of IFNβ, TNFα and IL–6. **(A-C)** WT Mouse Embryonic Fibroblasts (MEFs) were treated with specific TAK1 inhibitor 5Z-7-oxozeaenol (1 μM) 30 minutes prior to LTB_4_ (1 μM) treatment, IAV infection (0.5 m.o.i.) or IAV infection and LTB_4_ administration. **(D-F)** Mice were stimulated with MDP (10 μg/ml) alone or in combination with LTB_4_. Supernatants were collected 6 hours post-treatment and levels of **(A, D)** IFNβ, **(B, E)** TNFα and **(C, F)** IL–6 were determined by ELISA. Data are representative of three independent experiments. *p<0.05 as compared to indicated groups. n.d.: not detected.

To further validate that LTB_4_ targets TAK1 to potentiate NOD2 response to IAV infection, we have used an *in vitro* pharmacological approach to inhibit TAK1 activation since deletion of TAK1 gene is embryonically lethal [30]. To determine whether TAK1 is essential to LTB_4_ to potentiate NOD2-mediated response to IAV infection, we have pretreated mouse embryonic fibroblasts (MEFs) isolated from WT mice with TAK1 inhibitor prior infection with IAV and treatment with LTB_4_, and measured levels of phosphorylation of IRF3, IRF7 and translocation of NF-κB p65 subunit. LTB_4_ alone does not affect activation of IRF3 or IRF7 (Fig 6A and 6B) but seems to favor the translocation of the p65 subunit of NF-κB transcription factor in WT MEFs (Fig 6C). However, we observed an increased expression of p-IRF3 and p–65 in MEFs infected with IAV, which was markedly up-regulated when cells were costimulated with LTB_4_. Once again these effects were strongly reduced when cells were pretreated with TAK1 inhibitor (Fig 6A and 6C). In line with these results, pretreatment of WT MEFs with TAK1 inhibitor also prevents secretion of IFNβ, TNFα and IL–6 induced by a costimulation with IAV and LTB_4_ (Fig 7A–7C), as well as the enhanced production of TNFα and IL–6 in cells treated with MDP and LTB_4_ (Fig 7E and 7F). Together, these results clearly support that TAK1 is essential to the NOD2-IPS–1 axis in response to IAV infection and that TAK1 constitutes a central target for LTB_4_ to potentialize the NOD2-mediated responses. Thus, NOD2 can use either IPS–1 or RICK depending on the agonist recognized (viral RNA or MDP) to activate IRF3 or NF-κB, and TAK1 is essential to both axes to induce the production of IFNβ and cytokines.

## Discussion

Since its discovery, LTB_4_ has been seen as a central player of the inflammatory response. However, during the last decade, various reports have supported its potential to stimulate the innate response to control both bacterial and viral infection (reviewed in [8]). For example, LTB_4_ enhances the capacities of macrophages to ingest microbes and the capacity of neutrophils to secrete antimicrobial peptides [7,9]. LTB_4_ can also control viral infection, including IAV, by enhancing TLR-mediated innate response following recognition of viral components [10,11].

While initially identified as a host sensor of bacterial pathogen-associated molecular patterns (PAMPs), recent evidence demonstrated that NOD2 can also recognize ssRNA from respiratory viruses including IAV [16,23]. Because the actions of LTB_4_ on the innate response remain to be explored, the present study was initiated to define whether LTB_4_ may control IAV infection through its interactions with NOD2. Our results indicate that LTB_4_ treatment of IAV-infected mice increases viral clearance and improves lung architecture as compared to placebo-treated animals. These effects of LTB_4_ on viral clearance were also markedly affected in IPS–1^-/-^ and NOD2^-/-^ mice, supporting the contribution of NOD2 in LTB_4_ action. The control of IAV infection by LTB_4_ correlates with the activation of IRF3 and consequently with the secretion of IFNβ. In fact, while LTB_4_ alone does not induce the activation of IRF3, it strongly enhances phosphorylation of IRF3 in the presence of IAV. In addition to IRF3, treatment of IAV-infected mice with LTB_4_ was found to potentiate the translocation of NF-κB. While NF-κB is recognized to contribute to the activation of various cytokine genes, transactivating function of NF-κB was also reported to synergistically act on IRF3 for optimal production of type 1 IFN [27]. Thus, a defective activation of IRF3 and NF-κB in NOD2^-/-^ and IPS–1^-/-^ mice along with a reduced production of IFNβ and cytokines support the interactions of LTB_4_ with the NOD2 pathway in the induction of an antiviral response. However, we must not exclude that other sensors requiring activation of IPS–1 could be involved in the antiviral response against IAV. Indeed, RIG–1 is a RNA helicase which following recognition of IAV RNAs, recruits IPS–1 and consequently leads to the activation of IRF3 and production of type 1 IFNs [14,31–33]. In addition, NLRP3 is another innate receptor that senses IAV RNAs and requires IPS–1 for optimal activation of NLRP3 complex in response to viral infection [17,18,34–36]. Therefore, activation of these innate receptors following IAV infection of mice could compensate for the absence of functional NOD2.

Activation of NOD2 by bacterial components, including MDP, leads to the activation of NF-κB and MAPK via engagement of RICK. Interestingly, co-administration of LTB_4_ and MDP potentiates the production of TNFα and IL–6 in lungs of mice, an effect strongly reduced in NOD2^-/-^ mice. Therefore, these results reveal that LTB_4_ may interact with signaling molecules common to both NOD2-IPS–1 and NOD2-RICK axis. Contribution of TAK1 with NOD2 pathway was previously proposed when NOD2 was triggered by non-viral ligands such as MDP [28,29]. In this case, NOD2 oligomerization leads to RICK activation and consequently to TAK1 phosphorylation that results in engagement of MAPK and NF-κB. Similarly, our results clearly show that in response to IAV, TAK1 is essential to NOD2-dependent phosphorylation of IRF3 and type 1 IFN production, indicating that TAK1 is also an important element of the NOD2-IPS–1 axis. Moreover, this conclusion is confirmed by the treatment of MEFs with TAK1 inhibitor which impaired activation of IRF3 and production of IFNβ. This result is in agreement with a previous report showing that TAK1 has the capacity to generate phosphorylation of IRF3 [37]. Therefore, TAK1 plays a central role in the activation of the NOD2 pathway following its triggering by either a viral agonist or by a bacterial constituent.

We have previously reported that LTB_4_ can enhance response of TLR system to various ligands either by increasing expression of intracellular TLRs and by stimulating the activation of TAK1 [10,11]. NOD2 receptor expression in lung cells of IAV-infected mice was not affected by LTB_4_ treatment. However, we demonstrate that in addition to be an essential signaling event of the NOD2 cascade, TAK1 is also targeted by LTB_4_ to potentiate the activation of both IRF3 and NF-κB and consequently, the secretion of IFN and cytokines following triggering of NOD2. Innate receptors like TLRs and NLRs are interconnected via common components of their signaling pathway [38]. It is thus conceivable that LTB_4_ may also exert its effects on NLRP3 inflammasome since TAK1 has recently been proposed as a critical regulator of NLRP3 inflammasome activation [39]. Accordingly, ongoing experiments show that LTB_4_ potentiates IL-1β secretion in IAV infected mice and this effect is significantly abrogated in NLRP3^-/-^ animals (study in progress). LTB_4_ via its ability to activate TAK1 could also potentiate TAK1-associated triggering of NLRP3 and consequently enhance inflammasome complex formation to optimize host defense against viral infection. Thus, it is conceivable that LTB_4_ can trigger different pattern recognition receptor responses through its action on signaling molecules that intercept innate sensor pathways.

**Fig 8:**
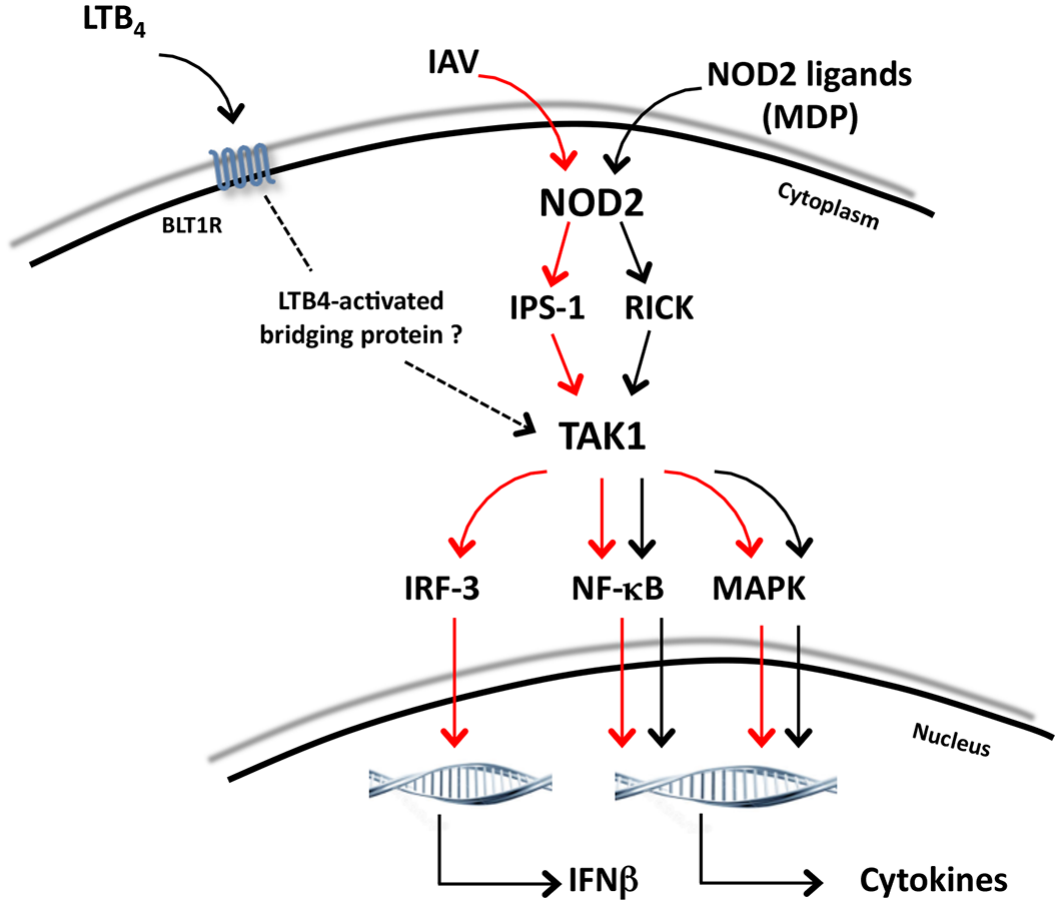
Schematic representation of LTB_4_ effects on NOD2 signaling. Following recognition of viral (IAV) or non-viral (MDP) ligands, NOD2 receptors signal through IPS–1 or RICK, respectively, to activate TAK1. Administration of LTB_4_ potentiates NOD2-mediated responses by acting on TAK1 (dotted arrow) at the branch of the adaptor protein IPS–1 in response to IAV infection (red arrows) or at the branch of the protein kinase RICK in response to MDP sensing (black arrows), which culminates in increased production of their respective inflammatory mediators. This boosting effect of LTB_4_ on TAK1 could require a bridging protein to induce an optimized NOD2-mediated innate immune response.

In light of our findings, we propose a mechanism through which LTB_4_ may potentiate NOD2-mediated innate immune response (Fig 8). LTB_4_, through its G protein-coupled receptor BLT1, potentiates NOD2-mediated responses by acting on TAK1 at the branch of IPS–1–IRF3-NF-κB/MAPK to induce production of IFNβ and cytokines when a virus like IAV is detected (red arrows), or at the branch of RICK-NF-κB/MAPK to induce secretion of cytokines when NOD2 is triggered by MDP (black arrows). However, direct interaction between LTB_4_ and TAK1 was not yet demonstrated and it is thus plausible that the effect of LTB_4_ on TAK1 (dotted arrow) can be mediated through a “LTB_4_-activated bridging protein” following NOD2 triggering. This aspect remains to be elucidated.

To conclude, our study highlights another mechanism activated by LTB_4_ to optimize innate defense against pathogens. By acting on TAK1 which is shared by different innate sensors, this makes LTB_4_ a powerful immunomodulatory molecule for the treatment of infection by a respiratory virus like IAV. Promising data obtained from previous clinical trials clearly showed that administration of LTB_4_ to human is safe, well tolerated and contributes to regulate innate response in both healthy volunteers and individuals infected with a respiratory virus [40,41]. Interactions of LTB_4_ with the immune system must be thoroughly investigated and could also provide insights for the elaboration of new therapeutic strategies to control viral infections.
